# Successful Laparoscopic Resection of 7 mm Ovarian Mature Cystic Teratoma Associated with Anti-NMDAR Encephalitis

**DOI:** 10.1155/2014/618742

**Published:** 2014-05-13

**Authors:** Masaru Hayashi, Emi Motegi, Koichi Honma, Nobuhide Masawa, Hideki Sakuta, Koichi Hirata, Yasushi Kaji, Ichio Fukasawa

**Affiliations:** ^1^Department of Obstetrics and Gynecology, Teine Keijinkai Hospital, Sapporo 006-8555, Japan; ^2^Department of Obstetrics and Gynecology, Dokkyo Medical University, Tochigi, Japan; ^3^Department of Anatomic and Diagnostic Pathology, Dokkyo Medical University, Tochigi, Japan; ^4^Department of Neurology, Dokkyo Medical University, Tochigi, Japan; ^5^Department of Radiology, Dokkyo Medical University, Tochigi, Japan

## Abstract

Anti-NMDAR (N-methyl-D-aspartate receptor) encephalitis is an immune-mediated encephalitis. It has been predominantly described in young women and is commonly associated with an ovarian teratoma. We report a case of anti-NMDAR encephalitis associated with a 7 mm ovarian teratoma that was completely resected by laparoscopic surgery. An 18-year-old woman suddenly presented with personality changes requiring her admission to the department of neurology. After that, she also showed involuntary movements, disturbance of consciousness, and central hypoventilation. As an abdominal image revealed the possibility of a right ovarian teratoma of 5 × 7 mm, a laparoscopic operation was performed. The macroscopic appearance of the right ovary did not show any abnormalities; nevertheless, we performed a partial resection of the right ovary, with reference to the image diagnosis, in order to spare the ovarian reserve. The 22 × 22 mm partially resected ovary contained an intact 5 × 7 mm cystic tumor. The pathological diagnosis was mature cystic teratoma with components of brain tissue. An anti-NMDAR-antibody test proved positive in both serum and cerebrospinal fluid 1 month after the surgery. From these results, she was diagnosed with anti-NMDAR encephalitis. By the administration of cyclophosphamide in addition to the operation, she recovered drastically without any of the symptoms shown before.

## 1. Introduction

Anti-NMDAR encephalitis is an immune-mediated encephalitis caused by N-methyl-D-aspartate receptor (NMDAR) antibody. The typical clinical feature of this encephalitis presents with neuropsychiatric symptoms, seizures, catatonia, unresponsiveness, dyskinesias, and central hypoventilation. It has been predominantly described in young females and is commonly associated with an ovarian teratoma. The production of anti-NMDAR antibody is activated by an immune response, usually triggered by a preceding infection. This anti-NMDAR antibody against antigens on the cell membrane of the nerve tissue in an ovarian teratoma induces an encephalitis arising from NMDAR dysfunction in the cerebral limbic system of a patient. The principle of the treatment for a patient with an ovarian teratoma is both immunotherapy and complete tumor removal as early as possible from the onset. We report here a case of anti-NMDAR encephalitis associated with a 7 mm ovarian teratoma that was completely resected by laparoscopic surgery with sparing of some ovarian reserve.

## 2. Case Report

**Figure 1:**
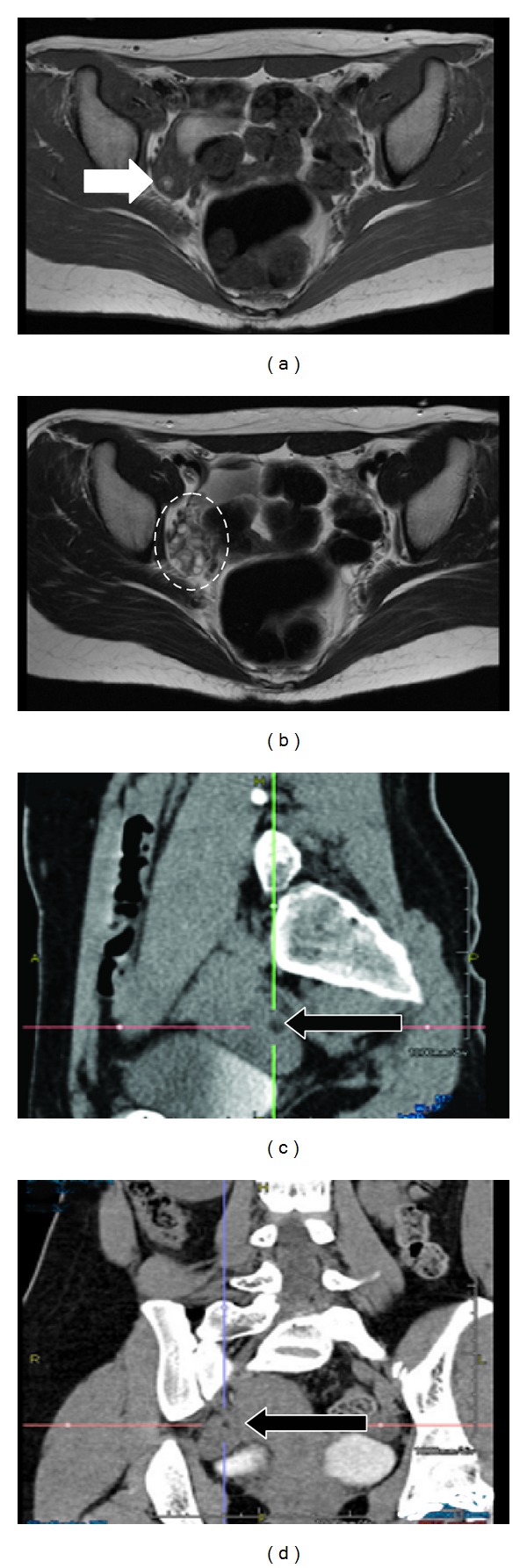
Image diagnosis. (a) T1-weighted transverse pelvic MRI; open arrow indicates a 5 × 7 mm high-intensity area in the right ovary that shows a fat component of the ovarian teratoma. (b) T2-weighted transverse pelvic MRI; broken-lined circle indicates the right ovary, which contains many antral follicles of the same size as the teratoma. ((c), (d)) Abdominal enhanced CT with 1 mm slice thickness in a sagittal plane (c) and in a coronal plane (d); closed arrow indicates a 5 × 7 mm low-density area in the right ovary that shows a fat component of the teratoma. The mean CT number was −62.5 HU.

An 18-year-old woman without a medical history showed headache and fever for a few days and then suddenly presented with personality changes with violent behavior and speech disabilities requiring her admission to the department of neurology in our hospital. A diagnostic image of her brain showed minimal changes such as mild brain edema, and cerebrospinal fluid (CSF) analysis showed lymphocyte pleocytosis suggesting inflammatory changes. As the first presumptive diagnosis was either viral encephalitis or autoimmune encephalitis, intravenous high dose steroids, acyclovir, and glycerol were administrated. Several days after her admission, she also showed involuntary movements, disturbance of consciousness, and central hypoventilation; she was managed by assisted respiration and medicated with midazolam and propofol. Since autoimmune encephalitis was the most possible diagnosis according to further examinations, plasma exchange treatment was also started, and the patient was introduced to our department for examination for a potential ovarian teratoma. Nevertheless, neither internal examination nor transvaginal ultrasonography detected an ovarian teratoma. T1-weighted pelvic magnetic resonance imaging (MRI) revealed a 5 × 7 mm fat intensity in the right ovary that showed the possibility of a teratoma (Figure 1(a)). T2-weighted pelvic MRI showed that a large number of similar-sized ovarian follicles surrounded the teratoma (Figure 1(b)). Initially, abdominal computed tomography (CT) with 10 mm slice thickness did not detect any fatty component in the right ovary. However, by referencing to the MRI findings, CT with 1 mm slice thickness eventually detected the fatty component in the right ovary. The mean CT number of that showed −62.5 HU (Figures 1(c) and 1(d)).

**Figure 2:**
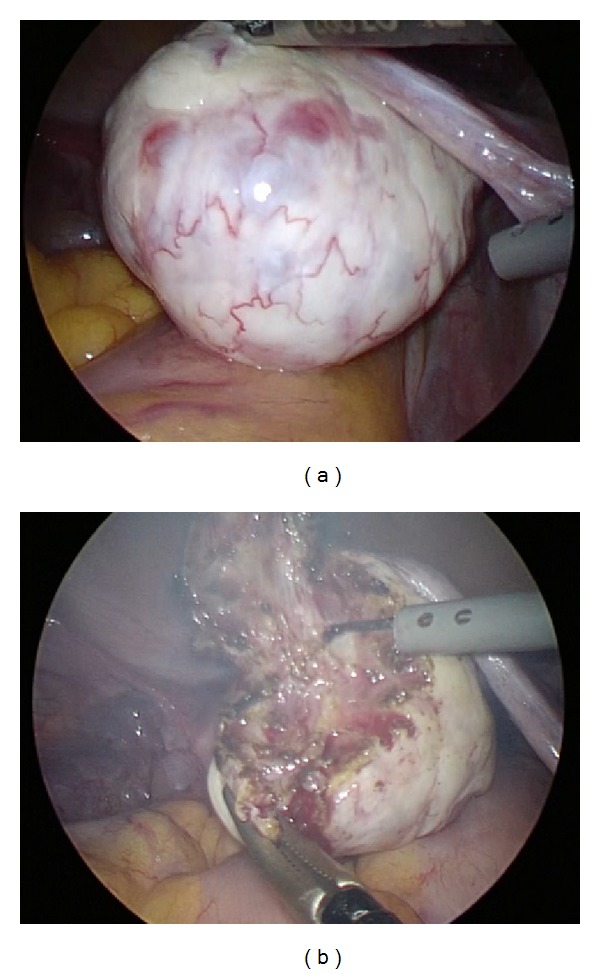
Laparoscopic surgery. (a) The macroscopic appearance of the right ovary did not show any abnormalities. (b) Partial resection of the right ovary was done by removing small pieces at a time starting from the most likely location of the teratoma, with reference to the diagnostic image where the teratoma was identified.

**Figure 3:**
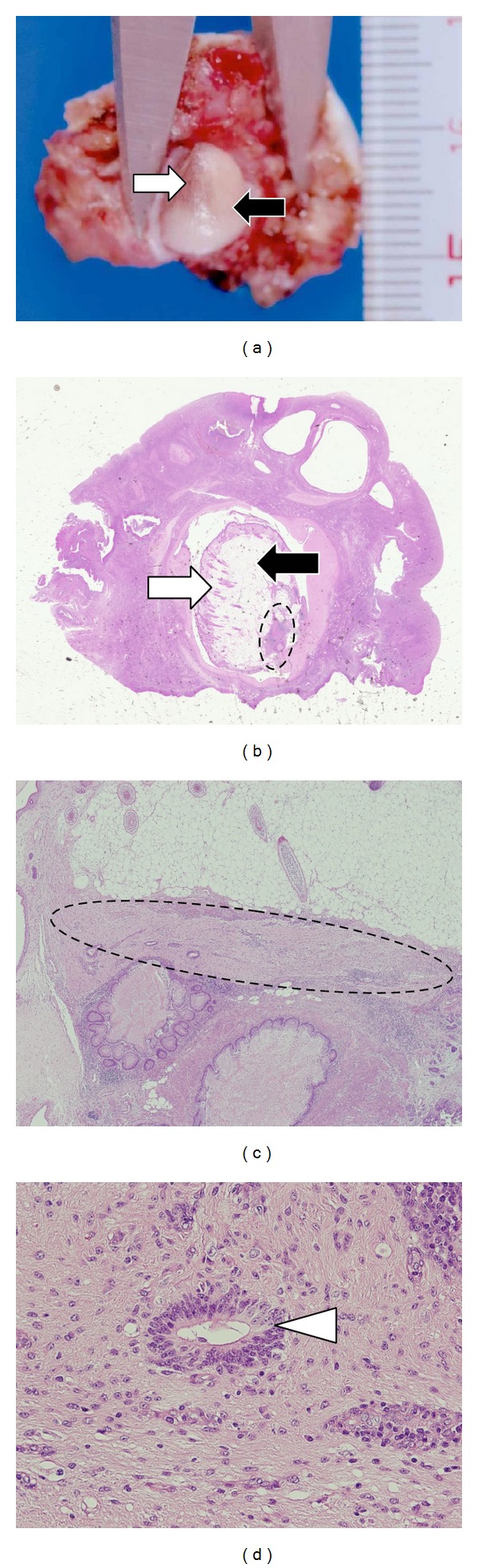
Pathological findings in the partially resected right ovary. (a) Gross appearance of the partially resected right ovary. There is a mature cystic teratoma in the ovary, measuring 5 by 7 mm. The open arrow indicates hair, and the closed arrow indicates fat. ((b), (c)) Low-power views of the histologic section of the mature cystic teratoma. The open arrow indicates hair, and the closed arrow indicates fat. The area circled by broken lines indicates brain tissue in the mature cystic teratoma ((b) H.E., ×1; (c) H.E., ×10). (d) High-power view of the mature cystic teratoma showing brain tissue. The open arrow head indicates a central canal-like structure (H.E., ×100).

After 18 days from the first visit to our hospital, the patient underwent a laparoscopic operation for complete resection of the right ovarian teratoma. The macroscopic appearance of the right ovary did not show any abnormalities (Figure 2(a)). We performed a partial resection of the right ovary by removing small pieces at a time starting from the most likely location of the teratoma according to the diagnostic image (Figure 2(b)). The first resected 22 × 22 mm ovarian piece contained an intact 5 × 7 mm cystic tumor in which we macroscopically identified fat and hair components (Figure 3(a)). The pathological diagnosis was mature cystic teratoma containing component of brain tissue (Figures 3(b)–3(d)).

An anti-NMDAR-antibody test was positive in both serum and cerebrospinal fluid 1 month after the surgery. From these results and her clinical course, the patient was diagnosed with anti-NMDAR encephalitis. After the surgery, clinical findings and symptoms are gradually improving. However, the recovery of her conscious level was slow and insufficient; 50 mg/m^2^ of cyclophosphamide in one course was administered 6 months after the operation. After 2 courses of cyclophosphamide, she showed drastic recovery, such as the complete elimination of involuntary movements and complete restoration of consciousness. Currently, while undergoing rehabilitation, she has recovered to the level of being able to ingest food by herself and walk with some help.

## 3. Discussion

Since Dalmau et al. described a new category of autoimmune encephalitis as anti-NMDAR encephalitis in 2007 [1], most neurologists and psychiatrists seem to recognize this disease, and many cases have been reported. Although this encephalitis presents very severe clinical symptoms and is potentially lethal, many patients recover by appropriate treatment [2]. The basic therapies for patients with a tumor involve both immunotherapy and early removal of the tumor [2–5]. Dalmau et al. reported that patients who had received early tumor treatment showed better outcomes than did those with late or no tumor treatment as well as those in whom no tumor was identified [5]. In this report, 58 of 98 patients (59.2%) had a tumor, and 56 of 58 patients with a tumor (96.5%) were women. Moreover, 53 of 56 female patients (94.6%) had an ovarian teratoma. Its median size was 6 cm, and the range was 1–22 cm. We think that some of the patients who were not identified with a tumor might have had it if more precise diagnostic imaging had been performed. Actually, in some patients, a trial oophorectomy showed ovarian teratoma [6, 7]. Furthermore, in one case an autopsy revealed a 7 mm ovarian teratoma [8]. Among reports in the English language literature our case was the smallest ovarian teratoma associated with anti-NMDAR encephalitis that was diagnosed before surgery or autopsy and subsequently completely removed by laparoscopic surgery. However, prior to an MRI examination we were unable to detect this 7 mm teratoma by transvaginal ultrasonography or abdominal CT with 10 mm slice thickness. If we are consulted about the diagnosis of an ovarian teratoma that is not detected by ordinary methods, we recommend using diagnostic imaging with slices as thin as possible, such as what we did in our case, because this encephalitis can occur even when a teratoma is very small.

We were concerned with whether we could identify the tiny teratoma in the right ovary by laparoscopy. If we had not found it, we would have decided to perform right salpingo-oophorectomy as a curative treatment for anti-NMDAR encephalitis. Simultaneously, we considered that an ovarian reserve should be spared as much as possible in such a young woman. Later, in this case two courses of cyclophosphamide were administered. Alkylating agents such as cyclophosphamide are known to be very toxic for an ovarian reserve, and the level of the original ovarian reserve is strongly related to the damage caused by chemotherapy [9–11]. For both of these reasons, we discussed the location of the teratoma in the right ovary with a radiologist in our hospital before surgery, and, as a result, we chose an operative method that consisted of partial resection of the right ovary by small pieces starting from the most likely location of the teratoma. This preoperative simulation with a radiologist was very helpful because the first resected ovarian piece contained the tiny teratoma. If this tiny teratoma had ruptured, the cystectomy might have become too difficult. Actually, the teratoma was surrounded by many antral follicles of the same size, as shown in Figure 1(b). The follicular fluid leaked out during dissection of the ovarian tissue, which made it more difficult to distinguish the teratoma. Asai et al. performed a laparoscopic cystectomy of a 10 mm ovarian teratoma in a 26-year-old woman with anti-NMDAR encephalitis; the location of the tumor was confirmed by a laparoscopic ultrasonography [12]. As we have no experience with laparoscopic ultrasonography, we do not know whether we could have identified the 7 mm teratoma located inside the ovary in our case by this method. Recently, when a teratoma is macroscopically identified, laparoscopic cystectomy is selected rather than oophorectomy, especially in young women. However, if it is difficult to identify an ovarian teratoma, we believe that it would be worthwhile to consider the method described in this report for both complete removal of ovarian teratoma and an ovarian reserve.

## References

[1] Dalmau J, Tüzün E, Wu H (2007). Paraneoplastic anti-N-methyl-D-aspartate receptor encephalitis associated with ovarian teratoma. *Annals of Neurology*.

[2] Dalmau J, Lancaster E, Martinez-Hernandez E, Rosenfeld MR, Balice-Gordon R (2011). Clinical experience and laboratory investigations in patients with anti-NMDAR encephalitis. *The Lancet Neurology*.

[3] Seki M, Suzuki S, Iizuka T (2008). Neurological response to early removal of ovarian teratoma in anti-NMDAR encephalitis. *Journal of Neurology, Neurosurgery and Psychiatry*.

[4] Iizuka T, Sakai F, Ide T (2008). Anti-NMDA receptor encephalitis in Japan: long-term outcome without tumor removal. *Neurology*.

[5] Dalmau J, Gleichman AJ, Hughes EG (2008). Anti-NMDA-receptor encephalitis: case series and analysis of the effects of antibodies. *The Lancet Neurology*.

[6] Boeck AL, Logemann F, Krauss T (2013). Ovarectomy despite negative imaging in anti-NMDA receptor encephalitis: effective even late. *Case Reports in Neurological Medicine*.

[7] Johnson N, Henry C, Fessler AJ, Dalmau J (2010). Anti-NMDA receptor encephalitis causing prolonged nonconvulsive status epilepticus. *Neurology*.

[8] Iizuka T (2009). Unique clinical features and pathophysiology of anti-NMDA receptor encephalitis. *Clinical Neurology*.

[9] Meirow D, Nugent D (2001). The effects of radiotherapy and chemotherapy on female reproduction. *Human Reproduction Update*.

[10] Cohen LE (2008). Cancer treatment and the ovary: the effects of chemotherapy and radiation. *Annals of the New York Academy of Sciences*.

[11] Kim CH, Jeon GH (2012). Fertility preservation in female cancer patients. *ISRN Obstetrics and Gynecology*.

[12] Asai S, Ishimoto H, Yabuno A, Asada H, Seki M, Iwata S (2011). Laparoscopic cystectomy of ovarian teratoma in anti-NMDAR encephalitis: 2 case reports. *Journal of Minimally Invasive Gynecology*.

